# The Pause before State Registration

**Published:** 1919-11-15

**Authors:** 


### THE MATRONS' AND SISTERS' DEPARTMENT.

### THE PAUSE BEFORE STATE REGISTRATION.

WITH the announcement that the Government Registration Bill has been read a first time in the House of Commons, a feeling of relief is experienced. Many nurses found it difficult to be patient in the waiting-time before the introduction of the promised measure of the Ministry of Health to regulate their profession. Nurses have waited a long time for legislation conceived in a wise and temperate spirit, in order to obtain the State recognition all agree is their due. And at the present moment, when conferences are being held and a Government Bill is before the House, it is natural that the spirit of reform should be working like yeast among nurses all over the kingdom. The wildest notions find acceptance with some members of the professions; the sanest arguments fail to satisfy. The one desire is to do something in a hurry and trust to good luck.

It is not only the hot-headed and discontented who are affected. All along the line, in every department of nursing, the coming of registration has been awaited with an almost desperate impatience. In truth nothing could be settled, no new beginnings made, until the new nursing constitution was promulgated. Of whatever nature it might prove to be, one thing was certain—that its provisions would have to be complied with. Hence a kind of lethargy seized nurses in regard to the many debatable points which have occupied their attention during the past two years. Why argue, they seemed to say, when all these matters will soon be taken out of the region of debate? Thus impatience and lethargy, two dispositions often found going hand in hand, have been holding nurses in bondage. And they are a bad preparation for the stirring times ahead.

It is well to remind ourselves that however comprehensive the new enactment for the State registration of nurses may be, it cannot cover the whole ground. It will not absolve the nursing profession from the duty of maintaining a strong professional body to look after the interests of nurses in general. If anyone imagines that the functions of the College of Nursing will be abrogated by the gaining of State recognition, they are likely to be speedily disillusioned. In every country where registration has found a home a powerful association has always sprung up side by side with it to clothe its dry bones with flesh and carry out for those who are qualified to be registered the numerous beneficial schemes which the State is not competent to undertake.

The College of Nursing started on its career when registration, for perfectly well-understood reasons, was under a cloud. The form under which this measure had been presented to nurses had been one they could not accept. Within a brief period the College was successful in demonstrating a sound and workable scheme for obtaining State recognition for nurses, and has been able to sweep out of the way the objections which had long stifled progress. But its success has been grounded on a broad basis. Not merely State registration but the protection and welfare of all nurses in every branch of their work is the College programme.

The call, then, in this waiting-time is to shake off lethargy and find a remedy for impatience in building the foundations of the College broad and deep now that the hour of registration strikes and the call for concerted action becomes imperative. The College may need to modify its programme in order to steer with the Ministry of Health. Some of the duties it has hitherto performed at great cost for the common good, will, under the Government registration scheme, be performed by the State at the public expense. But other of its duties will show an enormous quickening. New needs will arise. A hundred problems will confront nurses which none can solve but their own representatives working in their interests. Nurses must labour to fit themselves for these new responsibilities. They must learn to speak that they may educate those who still do not understand their own interests. They must practise in local centres the art of organisation and self-government. They must study in order to judge for themselves in the momentous matters which affect them. Above all they must set themselves in all earnestness to strengthen their professional association and make the best of all possible answers to their opponents by continually adding fresh thousands to the College.

